# Exogenous phthalanilic acid induces resistance to drought stress in pepper seedlings (*Capsicum annuum* L.)

**DOI:** 10.3389/fpls.2023.1156276

**Published:** 2023-09-27

**Authors:** Xiaopeng Lu, Qiong Wu, Keyi Nie, Hua Wu, Guangyou Chen, Jun Wang, Zhiqing Ma

**Affiliations:** ^1^ College of Plant Protection, Northwest A & F University, Yangling, China; ^2^ Provincial Center for Bio-Pesticide Engineering, Yangling, Shaanxi, China; ^3^ Institute of Water Conservancy and Soil Fertilizer, Xinjiang Academy of Agricultural Sciences/Northwest Oasis Water-saving Agriculture Key Laboratory, Ministry of Agriculture and Rural Affairs, Shihezi, Xinjiang, China

**Keywords:** malondialdehyde, photosynthesis, chlorophyll, soluble sugar, soluble protein, proline

## Abstract

Drought stress (DS) is one of the main abiotic negative factors for plants. Phthalanilic acid (PPA), as a plant growth regulator, can promote the growth and development of crops. In order to evaluate the ideal application concentration and frequency of PPA-induced drought resistance in pepper (*Capsicum annuum*) seedlings, the concentration of PPA was 133.3 mg·L^−1^; 200.0 mg·L^−1^; 266.7 mg·L^−1^, and some key indicators were investigated, including leaf wilting index (LWI), relative water content (RWC), and malondialdehyde (MDA). We found that the LWI and RWC in the PPA-applied pepper leaves under light drought stress (LDS) and moderate drought stress (MDS) were all elevated, while MDA contents were decreased. To better understand how PPA makes pepper drought resistant, we examined the photosynthetic characteristics, growth parameters, antioxidant activities, and osmotic substances in pepper seedlings treated twice with PPA at a concentration of 133.3 mg·L^−1^ under LDS, MDS, and severe drought stress (SDS). Results showed that PPA increased the chlorophyll, plant height, stem diameter, root-shoot ratio, and seedling index of pepper leaves under LDS, MDS, and SDS. The net photosynthetic rate (Pn), stomatal conductance (Gs), intercellular CO_2_ concentration (Ci), transpiration rates (Tr), and water-use efficiency (WUE) in the PPA-treated pepper leaves under LDS and MDS were improved, while their stomatal limitation (Ls) were reduced. PPA also boosted the activities of enzymatic antioxidants (superoxide dismutase, catalase, and peroxidase), as well as enhanced the accumulation of osmotic substances such as soluble sugar, soluble protein, and free proline in pepper leaves under LDS, MDS, and SDS. Thus, PPA can alleviate the growth inhibition and damage to pepper seedlings caused by DS, and the PPA-mediated efficacy may be associated with the improvement in PPA-mediated antioxidant activities, Pn, and accumulation of osmotic substances.

## 1 Introduction

Plants are susceptible to various abiotic factors throughout their life cycle, including salinity, drought, high and low temperature, heavy metal, hypoxia, and high wind stresses (Etesami, 2018). Drought stress (DS) is considered to be among the most critical and devastating abiotic stresses (Grillakis, 2019). The physiological and biochemical processes of plants, such as photosynthesis, respiration, secondary metabolites, and enzyme activities, are severely suppressed under DS (Okunlola et al., 2017), resulting in a reduction in yield (Hussain et al., 2018; Leng and Hall, 2019). Especially in recent years, global climate change has exacerbated the frequency and severity of DS (Harrison et al., 2014; Liu et al., 2019). Meanwhile, it has been reported that the world’s population is expected to reach approximately 9.8 billion by 2050 (Mphande et al., 2020). Therefore, it is necessary to investigate approaches to overcome the yield losses brought on by DS in order to avoid a global food shortage as food demand rises.

Irrigation can mitigate the negative impact of drought stress on crops, but this measure is difficult to implement in most cases due to water resource constraints (Leng and Hall, 2019). The application of exogenous compounds is a common practice to stimulate the drought resistance of crops, such as plant growth regulators (PGR) (Chen et al., 2018), nutrients (Etesami and Jeong, 2018; Shehzad et al., 2020), and phytohormones or analogues (Anjum et al., 2016). For example, PGRs, such as spermidine (Li et al., 2018), and 24-epibrassinolide (EBL) etc. (Shahzad et al., 2018), are a potent tool for sustainably mitigating drought stress in many plants. Phytohormones or analogues are also a promising practical strategy to enhance crop drought resistance, such as brassinolide (Anjum et al., 2011), brassinosteroids (Krishna et al., 2017), jasmonic acid (Farhangi-Abriz and Ghassemi-Golezani, 2019), methyl jasmonate (Anjum et al., 2016), salicylic acid (Sharma et al., 2018), cytokinin (Egamberdieva et al., 2017), melatonin (Sharma et al., 2020), and gibberellin (Gaion et al., 2018).

Phthalanilic acid [2- (phenyl carbamoyl) benzoic acid], also known as N-phenyl-phthalamic acid (PPA), is a PGR developed by the Neviki Research Institute of Hungary in 1982 (Zhao et al., 2014). It can improve the stigma vitality, pollination, setting fruit, and yield of many fruit trees without causing any phytotoxicity (Racsko, 2004; Khadivi-Khub and Nosrati, 2013). Our previous investigations showed that PPA significantly increased the yield of pepper (*Capsicum annuum*) fruits as well as enhanced the stress resistance of pepper plants in field conditions (Zhang et al., 2017; Wu et al., 2018; Zhang et al., 2018), especially in the typical arid and semi-arid regions of northwest China, where drought often occurs due to the frequent shortage of water resources (Yang et al., 2018). Thus, we believed that PPA may stimulate resistance to DS in peppers, thereby reducing the negative effects of DS on peppers. However, the role of PPA in mitigating DS-induced damage is only partially understood and merits further investigation.

In general, the plants in response to drought stress cause changes in several important markers, including malondialdehyde (MDA), proline, and the activity of antioxidant enzymes such as superoxide dismutase (SOD), catalase (CAT), and peroxidase (POD) (Beyaz, 2019; Beyaz, 2022). These markers are often used as the basis for drought stress studies of pepper (Kim et al., 2022). Therefore, to elucidate the drought-resistance effect of PPA on pepper (*C. annuum*) seedlings, the content of these markers was measured in this study. Besides, this work also made in-depth studies on other characteristics of pepper, such as the leaf wilting index, relative water content, photosynthetic characteristics, growth parameters, and osmotic substances.

## 2 Materials and methods

### 2.1 Plant material and chemicals

Pepper (*C. annuum*, Shijihong) seeds were obtained from the Horticulture College of Northwest A & F University. PPA 20% soluble liquid (SL) was provided by Shaanxi Sunger Road Bio-science Co., Ltd. Trichloroacetic acid, coomassie brilliant blue G-250, thiobarbituric acid, and other reagents were provided by Aladdin™ (Shanghai, China).

### 2.2 Experimental design

The pepper seeds were sown in the plug trays with 32 holes (the top diameter of 6 cm, the bottom diameter of 3.0 cm, and 5.5 cm depth) filled with the nutritive soil (garden soil: compost: humus = 1:1:1, v/v/v). The trays were then placed in a temperature-controlled climatic chamber (L:D = 12:12 h, RH = 60%-70%, T = 25 ± 5 °C) to continue cultivation. At the five-leaf stage, the pepper seedlings were treated with PPA (133.3 mg·L^−1^; 200.0 mg·L^−1^; 266.7 mg·L^−1^) at 8:00 - 9:00 am by using a small manual sprayer. Water was applied as a control. The number of PPA treatments was also thought to be a factor. PPA was sprayed again at a two-day interval. Then, the pepper seedlings were transplanted to new trays. To control soil moisture in the new trays, the nutritive soil was dried to a constant weight in an oven at 105 °C for 8 h, and then mixed with different proportions of water (mass ratio). In this experiment, four drought conditions were set according to the soil relative water (SRW) content, including (1) the normal group representing that the SRW content was maintained between 70% and 80%, (2) the light drought stress (LDS) group (SRW: 50% ~ 60%), (3) the moderate drought stress (MDS) group (SRW: 40%~50%), and (4) the severe drought stress (SDS) group (SRW: 30% ~ 40%). The leaf wilting index, relative water content, and malondialdehyde content of pepper seedlings under LDS and MDS for 15 days were measured to evaluate the ideal application concentration and frequency of PPA.

To further clarify the mechanism of PPA causing drought resistance in pepper, the morphological indicators, photosynthetic parameters, antioxidant activities, and osmotic substances of pepper seedlings treated twice with PPA at a concentration of 133.3 mg·L^−1^ were further measured on days 1, 3, 7, 11, 15 (LDS and MDS) or on days 1, 3, 5, 7, 10 (SDS). Each drought condition was repeated three times, and each repetition contained 30 pepper seedlings.

### 2.3 Morphological indicators of pepper seedlings

The plant height and stem diameter were measured five times using a ruler and vernier caliper, respectively (Xiong et al., 2016). The pepper seedlings were washed with deionized water and dried naturally to determine the dry weight of the whole plant, including the dry weight of underground and above-ground parts. Each treatment was repeated three times, and five pepper seedlings were randomly selected from each repetition. The root-shoot ratio, seedling index, and leaf wilting index were calculated according to the following formulas:


Root-shoot ratio(RSR)=DUW/DAW

Seedling index (SI)=(SD/PH+DUW/DAW)× DWW

Leaf wilting index (LWI)=(1–NWL/NTL)×100%

Where DWW, DUW, and DAW represent the dry weight of the whole plant (g), the dry weight of underground parts (g), and the dry weight of above-ground parts (g), respectively. SD and PH are stem diameter (cm) and plant height (cm), respectively. NWL and NTL represent the number of wilted leaves and the number of total leaves, respectively.

### 2.4 Relative water content

Relative water content was measured according to the method of Liang et al. (2018). Five leaves were randomly collected from each plant, and the fresh weight (FW) of the leaves was recorded. The leaves were immersed in distilled water for 24 h to further measure the turgid weight (TW). Finally, the leaves placed in an oven were roasted at 105 °C for 30 min, and then dried at 80 °C to a constant weight, and the dry weight (DW) was determined.


Relative water content (RWC)=(FW–DW)/(TW–DW)×100%

### 2.5 Chlorophyll content

Chlorophyll content was determined according to the method of Min et al. (2019), with some modifications. Ten fresh leaves were collected from each plot and washed as test samples. The samples (0.3 g) were then ground to homogenate in a mortar with quartz sand, calcium carbonate powder, and 5.0 ml of pure acetone. The extracting reaction was performed by adding 5.0 mL of 80% (w/v) acetone to the homogenate for 10 min under dark conditions. The extraction solution was centrifuged using the Centrifuge 5424 (Eppendorf AG, Germany) at 20, 000 g for 30 min at 4 °C. Supernatant was collected, and its absorbance was recorded by the UV­3310 spectrophotometer (Hitachi, Japan) at 663 nm and 645 nm, respectively. There were three replicates for each treatment. The chlorophyll content (chlorophyll *a* and chlorophyll *b*) was calculated using the equation of Min et al. (2019). The results are expressed on a dry weight basis (mg·g^−1^ FW).

### 2.6 Photosynthetic parameters

The photosynthetic parameters, including net photosynthesis rate (Pn), transpiration rate (Tr), stomatal conductance (Gs), intercellular CO_2_ concentration (Ci), and atmospheric CO_2_ concentration (Ca), were measured using a portable photosynthesis instrument (PP-Systems, MA, USA) on a sunny day between 9:00 and 11:00 a.m. The light intensity was set to 100.0 μmol·m^−2^·s^−1^. The flow rate of the air with 60%­70% relative humidity was maintained at 500.0 μmol·s^−1^. The leaf temperature was 25 ± 1.5 °C, and the CO_2_ concentration was set at 400 μmol·mol^−1^. The instantaneous water-use efficiency (WUE) was calculated as Pn/Tr. Stomatal limitation (Ls) was calculated as Ls = 1 – Ci/Ca. Among them, the fully expanded uppermost leaf (from the top of the main stem) of each plant was selected for the photosynthetic parameter’s measurement. Each treatment was repeated three times, and five plants were randomly selected from each repetition.

### 2.7 Malondialdehyde content

Malondialdehyde (MDA) content was measured as described by Goñi et al. (2018) with minor modifications. Briefly, the sample of fresh leaf (1.0 g) was ground to homogenate. The homogenate added 10.0 mL of 20% (w/v) trichloroacetic acid (TCA) was centrifuged at 10, 000 g for 10 min at 4 °C. Supernatant (2.0 mL) was incubated with 2.0 mL of thiobarbituric acid (0.5%, w/v) at 95 °C for 15 min. After centrifugation again, the absorbance of the supernatant was recorded at 450 nm, 532 nm, and 600 nm. The MDA content was calculated according to the following equations,


$$\text{MDA }(\text{nmol MDA}\cdot {\text{g}}^{-1}\text{FW})=\;[({\text{OD}}_{532}–{\text{OD}}_{600})\times 6.452–0.559\times {\text{OD}}_{450}]\times \text{VE}/\left(\text{VEM}\times \text{W}\right)$$

Where VE means the total volume of the extraction solution (mL); VEM is the total volume of the extraction solution for measurement (mL); W represents the fresh weight of the sample (g).

### 2.8 Free proline content

The procedure of Bates et al. (1973) with some modifications was used for the measurement of free proline. Briefly, leaves (0.5 g) were homogenized in 10.0 mL of 3% (w/v) sulfosalicylic acid. The homogenate was centrifuged at 15, 000 × *g* for 30 min at 4 °C. Then, its supernatant (2.0 mL) was mixed with acidic ninhydrin (2.0 mL) and acetic acid (2.0 mL) and boiled for 40 min. After cooling at room temperature, the mixture was extracted with toluene (4.0 mL), and the absorbance of the toluene extract was read at 520 nm. The free proline content was calculated by the standard curve method and expressed as mg·g^−1^ FW.

### 2.9 Extraction and determination of antioxidant enzymes

Antioxidant enzyme activities, including superoxide dismutase (SOD), catalase (CAT), and peroxidase (POD), were determined following the protocol of Shehzad et al. (2020) and expressed as U·g^−1^ FW. Firstly, leaves (0.5 g) were fully homogenized with 2.0 mL of pre-cooled phosphate buffer (50 mM, pH 7.8) and 7.0 mL of 1.0% (w/w) polyvinylpyrrolidone. The homogenate was then centrifuged at 8, 000 × *g* for 15 min at 4 °C to collect supernatant for enzyme analysis. The absorbance was monitored every 20 s by a UV-3310 spectrophotometer (Hitachi, Japan) at 240 nm wavelength. The CAT activity was evaluated by measuring the decomposition of H_2_O_2_. The POD activity was assessed by monitoring the absorbance at 470 nm using guaiacol. To assay the SOD activity, the absorbance was measured at 570 nm after 20 min of chromogenic reaction. All enzymes’ activities were expressed as units U·g^−1^·FW.

### 2.10 Soluble sugar content

Soluble sugars were determined according to the method described by Du et al. (2020). The fresh leaves (0.5 g) were extracted in the boiling water bath for 30 min. Its remaining residue was extracted twice more, and the extraction solutions were combined in a volumetric flask (100.0 mL). Then, the test tube with extraction solution (0.5 mL) added cocktail consisting of 0.5 mL of anthrone-ethyl acetate reagent, 5.0 mL of concentrated sulfuric acid, and distilled water (1.5 mL). After shaking, the test tube was immediately put into the boiling water bath for 10 min. After cooling, absorbance was determined three times by the UV-3310 spectrophotometer (Hitachi, Japan) at 620 nm wavelength. The soluble sugar content was calculated by a standard curve (glucose) and expressed as mg·g^−1^ FW.

### 2.11 Soluble protein content

Soluble protein was measured using the UV-3310 spectrophotometer (Hitachi, Japan) at 595 nm. Briefly, leaves (0.5 g) were ground to homogenate with 5.0 mL of distilled water, and then the homogenate was centrifuged at 12, 000 × *g* for 20 min at 4 °C. Supernatant (1.0 mL) was collected and added to 5.0 mL of Coomassie Brilliant Blue G-250 solution in the test tube at 30 °C for 30 min for the soluble protein determination. The absorbance of the reaction solution was measured with a spectrophotometer at 595 nm. Bovine serum albumin was used as a standard to quantitatively analyze the content of soluble protein, which was expressed as mg·g^−1^ FW (Zou et al., 2017).

### 2.12 Statistical analysis

All data were analyzed with the SPSS 20.0 statistics package (Ver. 22.0, IBM, USA). A one-way analysis of variance (ANOVA) followed by Duncan’s multiple range test (*P*< 0.05) was used to assess the differences between means. All graphics were drawn using Origin version 8.1.

## 3 Results

### 3.1 Effect of PPA on the LWI, RWC and MDA of pepper seedlings under DS

The effects of PPA on pepper plants under LDS or MDS situations are shown in **Tables 1**, **2**. The pepper leaves under LDS did not become wilted, so the LWI value did not exist. Compared with the control, the LWI values were increased in the PPA-treated pepper seedlings under MDS, especially with the notable increase rate of 23.38% on the 133.3 mg·L^−1^ of PPA treatment (sprayed twice). The PPA also raised the RWC of pepper leaves. Notably, in comparison with the control, RWC in the pepper leaves treated with PPA (133.3 mg·L^−1^) twice was significantly (*P*< 0.05) increased by 21.47% and 9.32% under LDS and MDS, respectively. MDA content in the PPA-treated pepper leaves under drought decreased in comparison with the control. In particular, PPA that was applied two times at a concentration of 133.3 mg·L^−1^ observably (*P*< 0.05) reduced the MDA content by 44.42% and 42.30% under LDS and MDS, respectively. According to the changes in LWI, RWC, and MDA, the PPA-pretreated pepper seedlings had preferable resistance to DS. Therefore, the effects of PPA-induced resistance to DS were further researched in pepper seedlings.

**Table 1:** Effect of PPA on pepper (C. annuum) seedlings under light drought stress.

Treatment ^†^	Concentration (mg·L^−1^)	Number of applications	RWC (%)	MDA content (mmol·g^−1^ FW)
Control	—	—	73.95 ± 1.43d	9.41 ± 0.65a
PPA	133.3	1	82.15 ± 1.14b	6.02 ± 0.53c
		2	89.83 ± 1.45a	5.23 ± 0.23d
	200.0	1	81.72 ± 1.23b	5.40 ± 0.35cd
		2	77.95 ± 1.14c	5.93 ± 0.48cd
	266.7	1	76.04 ± 0.54c	6.50 ± 0.23c
		2	75.78 ± 0.90cd	8.40 ± 0.25b

† PPA is an abbreviation of phthalanilic acid.

Data are the average of three replications (n = 3) and represented as mean ± standard deviation. Values followed by different small letters in the same column are significantly different at P< 0.05.

RWC represents the relative water content of pepper leaves. MDA is the malondialdehyde content in pepper leaves.

**Table 2:** Effect of PPA on pepper (C. annuum) seedlings under moderate drought stress.

Treatment ^†^	Concentration (mg·L^−1^)	Number of applications	LWI (%)	RWC (%)	MDA content (mmol·g^−1^ FW)
Control	—	—	61.11 ± 4.62bc	62.22 ± 1.59d	13.97 ± 0.83a
PPA	133.3	1	74.14 ± 3.18a	73.69 ± 1.19b	11.01 ± 0.86b
		2	75.40 ± 4.87a	68.02 ± 2.19c	8.06 ± 0.23c
	200.0	1	68.26 ± 2.75ab	68.27 ± 1.33c	10.64 ± 0.95b
		2	69.56 ± 2.55ab	77.40 ± 1.52a	11.50 ± 0.96b
	266.7	1	67.26 ± 2.85b	66.68 ± 1.19c	10.98 ± 0.25b
		2	63.49 ± 3.50b	65.99 ± 1.43c	11.34 ± 1.13b

^†^PPA is an abbreviation of phthalanilic acid.

Data are the average of three replications (n = 3) and represented as mean ± standard deviation. Values followed by different small letters in the same column are significantly different at P< 0.05.

LWI means the leaf wilting index of pepper leaves.

### 3.2 Effect of PPA on morphological indices of pepper seedlings under DS

The PPA-pretreated pepper seedlings exhibited obvious advantages over water-pretreated pepper seedlings in plant height, root-shoot ratio, and seedling index under three drought conditions (**Table 3**). Among them, the seedling index increased by 20.54% (LDS), 21.29% (MDS), and 23.20% (SDS), respectively. The stem diameter was increased by 1.18 times under SDS. However, the stem diameter was not affected by PPA under LDS or MDS.

**Table 3:** Effect of PPA on morphological indicators of pepper (C. annuum) seedlings under drought stress.

Treatment ^†^	Plant height (cm)	Stem diameter (cm)	Root-shoot ratio	Seedling index
Light drought stress				
Control	13.492 ± 0.128	2.103 ± 0.033	0.230 ± 0.010	0.370 ± 0.021
PPA	13.958 ± 0.170*	2.145 ± 0.015	0.243 ± 0.010*	0.446 ± 0.017*
Moderate drought stress				
Control	13.258 ± 0.138	0.208 ± 0.030	0.181 ± 0.007	0.310 ± 0.009
PPA	13.667 ± 0.092*	0.212 ± 0.018	0.212 ± 0.09*	0.376 ± 0.013*
Severe drought stress				
Control	13.285 ± 0.104	1.735 ± 0.036	0.094 ± 0.008	0.250 ± 0.012
PPA	14.158 ± 0.325*	2.043 ± 0.048*	0.106 ± 0.009	0.308 ± 0.014*

† PPA was twice applied to pepper seedlings at a concentration of 133.3 mg•L−1 before drought stress.

The data are presented as the mean value ± standard deviation (n = 3), and asterisk denotes significant difference to corresponding control at P< 0.05.

### 3.3 Effect of PPA on photosynthetic parameters of pepper seedlings under DS

**Figure 1:**
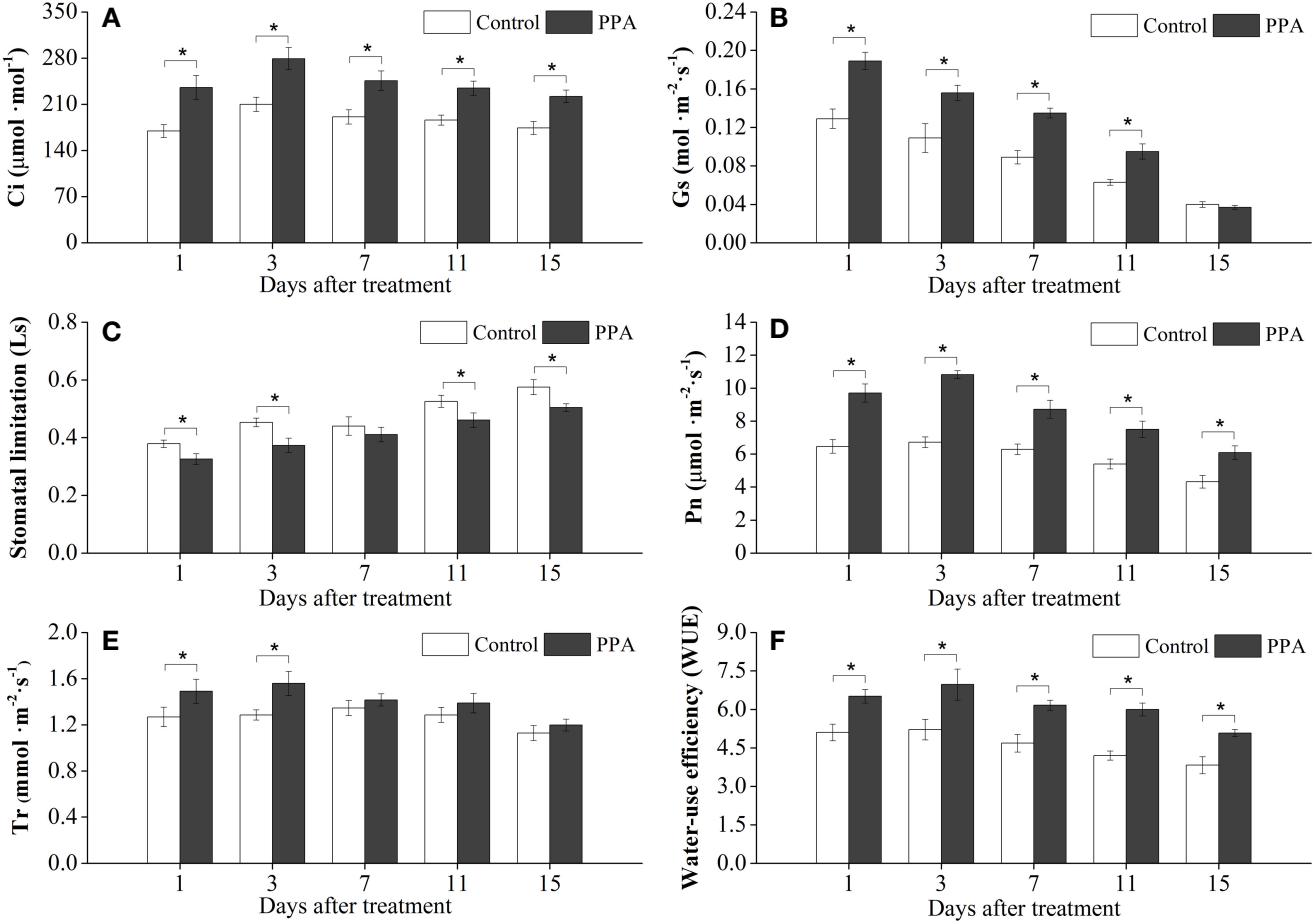
Effects of PPA on the intercellular CO_2_ concentration (Ci; **A**), stomatal conductance (Gs; **B**), stomatal limitation (Ls; **C**), net photosynthesis rate (Pn; **D**), transpiration rates (Tr; **E**), and water-use efficiency (WUE; **F**) of pepper (*Capsicum annuum*) leaves under light drought stress (LDS). LDS means that pepper seedlings were maintained the SRW content of 50% - 60%. Control means that pepper seedlings were treated with water before drought stress and maintained the soil relative water (SRW) content of 70% - 80%. Bars represent means ± SD of three replications, and asterisk denotes significant difference to corresponding control at *P*< 0.05. Phthalanilic acid (PPA) was twice applied to pepper seedlings at a concentration of 133.3 mg·L^−1^ before drought stress. The same as **Figures 2**–**6**.

As illustrated in **Figure 1**, the exogenous application of PPA improved the net photosynthetic rate (Pn), transpiration rates (Tr), stomatal conductance (Gs), intercellular CO_2_ concentration (Ci), and water-use efficiency (WUE) on the pepper seedling leaves under LDS but reduced the values of stomatal limitation (Ls). Among them, the PPA-treated pepper seedlings showed remarkable (*P*< 0.05) increases in Pn (38.65% - 61.04%), Ci (27.43% - 38.81%), and WUE (20.61% - 33.63%) during the LDS period over control. The Gs values were significantly (*P*< 0.05) higher than those of the control, with the increase rates ranging from 33.12% to 51.69% by day 11, but there were no significant changes for the Gs value on day 15. The Ls values were markedly (*P*< 0.05) lower than those of control by a range of 12.53% to 17.66%, except on the 7^th^ day. The transpiration rates (Tr) were significantly (*P*< 0.05) improved by 17.59% (on day 1) and 21.23% (on day 3) over control under LDS (**Figure 1**).

**Figure 2:**
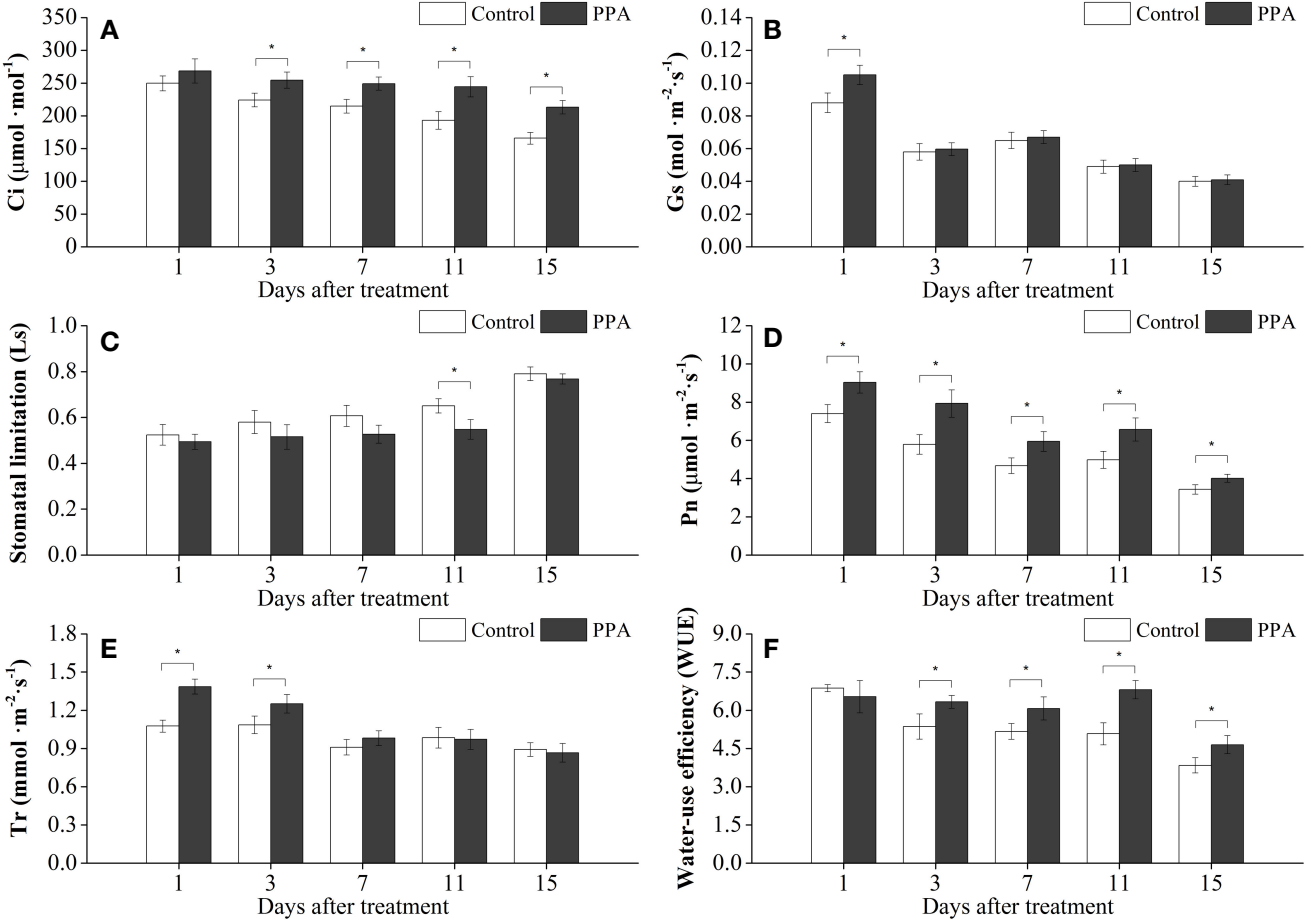
Effects of PPA on the Ci **(A)**, Gs **(B)**, Ls **(C)**, Pn **(D)**, Tr **(E)**, WUE **(F)** of pepper (*C. annuum*) leaves under moderate drought stress (MDS). MDS represents that pepper seedlings were kept the SRW content of 40% - 50%. Asterisk denotes significant difference to corresponding control at P < 0.05.

Similarly, PPA enhanced the Pn, Ci, and WUE on the pepper leaves under MDS, with marked (*P*< 0.05) increase rates of 17.23% - 36.94% (from day 1 to day 15), 13.43% - 28.53% (from day 3 to day 15), and 17.44% - 33.92% (from day 3 to 15) in comparison with control (**Figure 2**); and the Gs value was observably (*P*< 0.05) improved by 19.32% on day 1. PPA also raised the Tr values of pepper leaves during the MDS period; especially compared with the control, the Tr values were observably (*P*< 0.05) increased by 28.81% (on day 1) and 15.19% (on day 3) (**Figure 2**). However, the values of Ls were decreased by PPA under MDS for 15 days, with a notable (*P*< 0.05) reduction of 15.82% (on day 11) over control (**Figure 2**).

**Figure 3:**
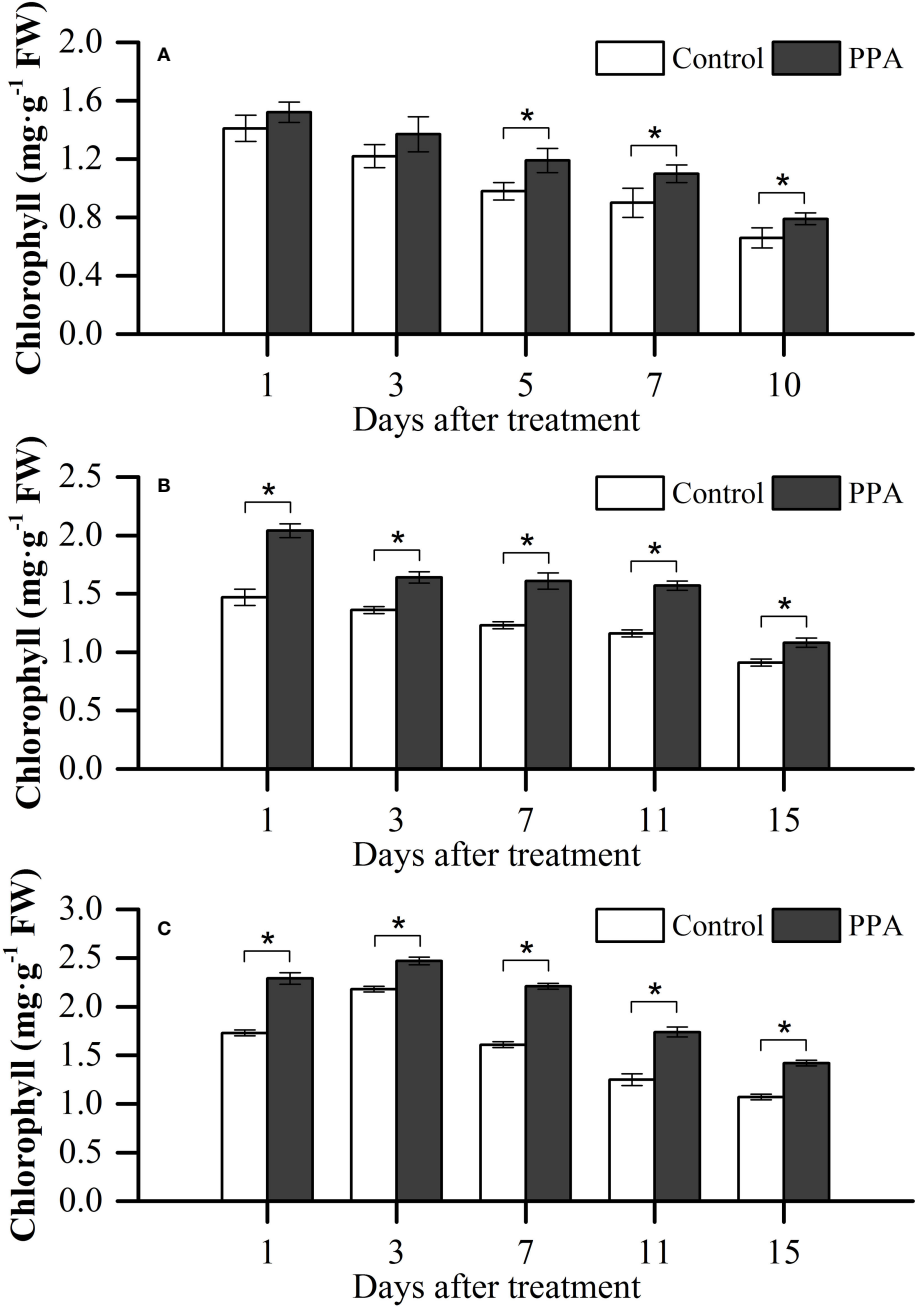
Effect of PPA on the chlorophyll of pepper (*C. annuum*) leaves under severe drought stress (SDS; **A**), moderate drought stress (MDS; **B**), and light drought stress (LDS; **C**). Among them, SDS symbolizes that pepper seedlings were provided the SRW content of 30% - 40%. Asterisk denotes significant difference to corresponding control at P < 0.05.

The chlorophyll contents of PPA-treated peppers were higher than those of non PPA-treated peppers under SDS, with significant (*P*< 0.05) increases ranging from 19.69% (on day 5) to 22.22% (on day 10) (**Figure 3A**). PPA had significant effects on chlorophyll contents within the MDS period, with the increase rate ranging between 18.68% and 38.78% (**Figure 3B**). As shown in **Figure 3C**, in comparison with the control, the chlorophyll contents in PPA-applied pepper leaves were obviously (*P*< 0.05) increased by between 13.30% and 39.20% under the LDS situation for 15 days.

### 3.4 Effect of PPA on antioxidant enzyme activities in pepper seedlings under DS

**Figure 4:**
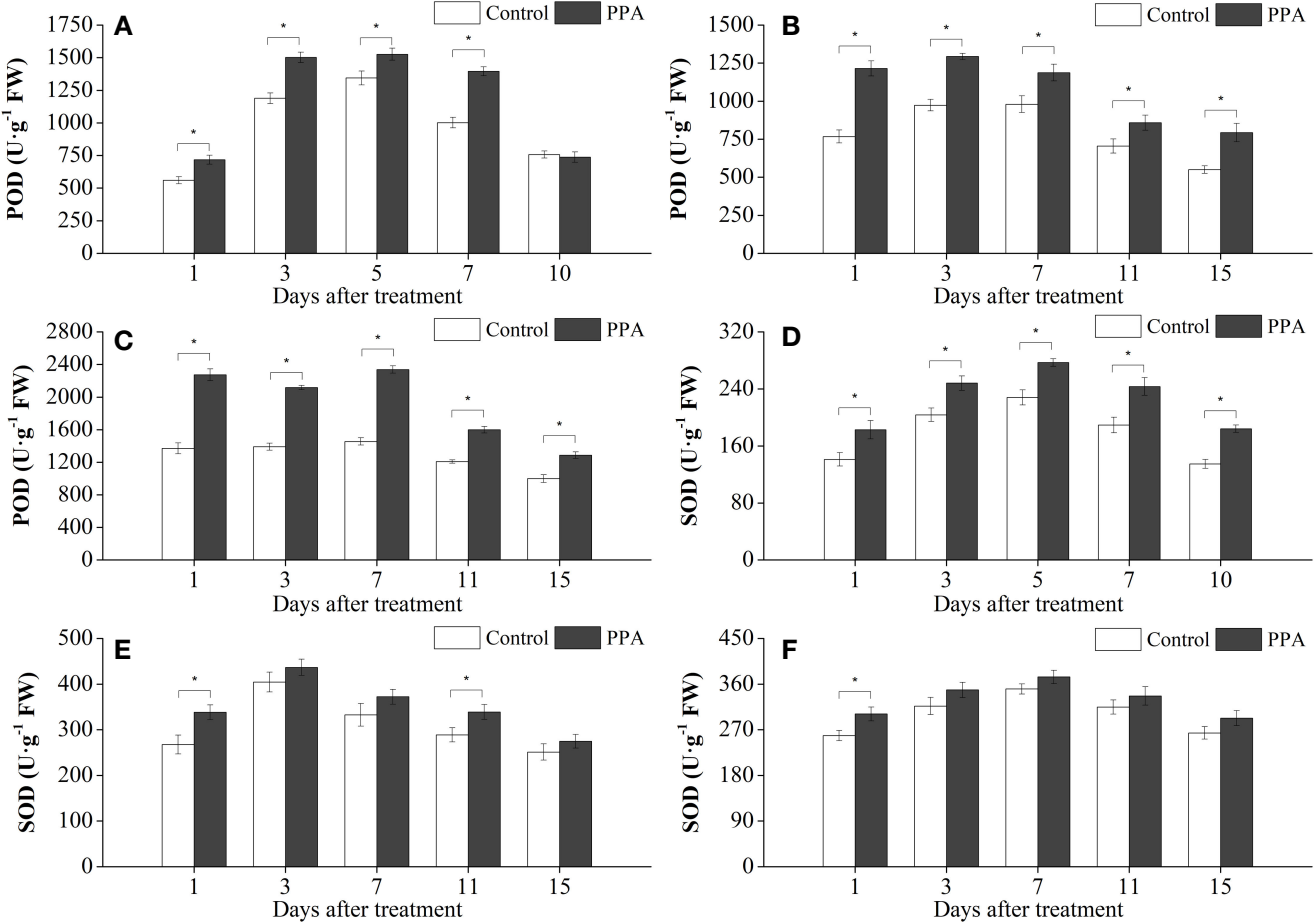
Effect of PPA on POD (**A**, SDS; **B**, MDS; **C**, LDS) and SOD (**D**, SDS; **E**, MDS; **F**, LDS) in pepper (*C. annuum*) leaves under drought stress. Asterisk denotes significant difference to corresponding control at P < 0.05.

**Figure 5:**
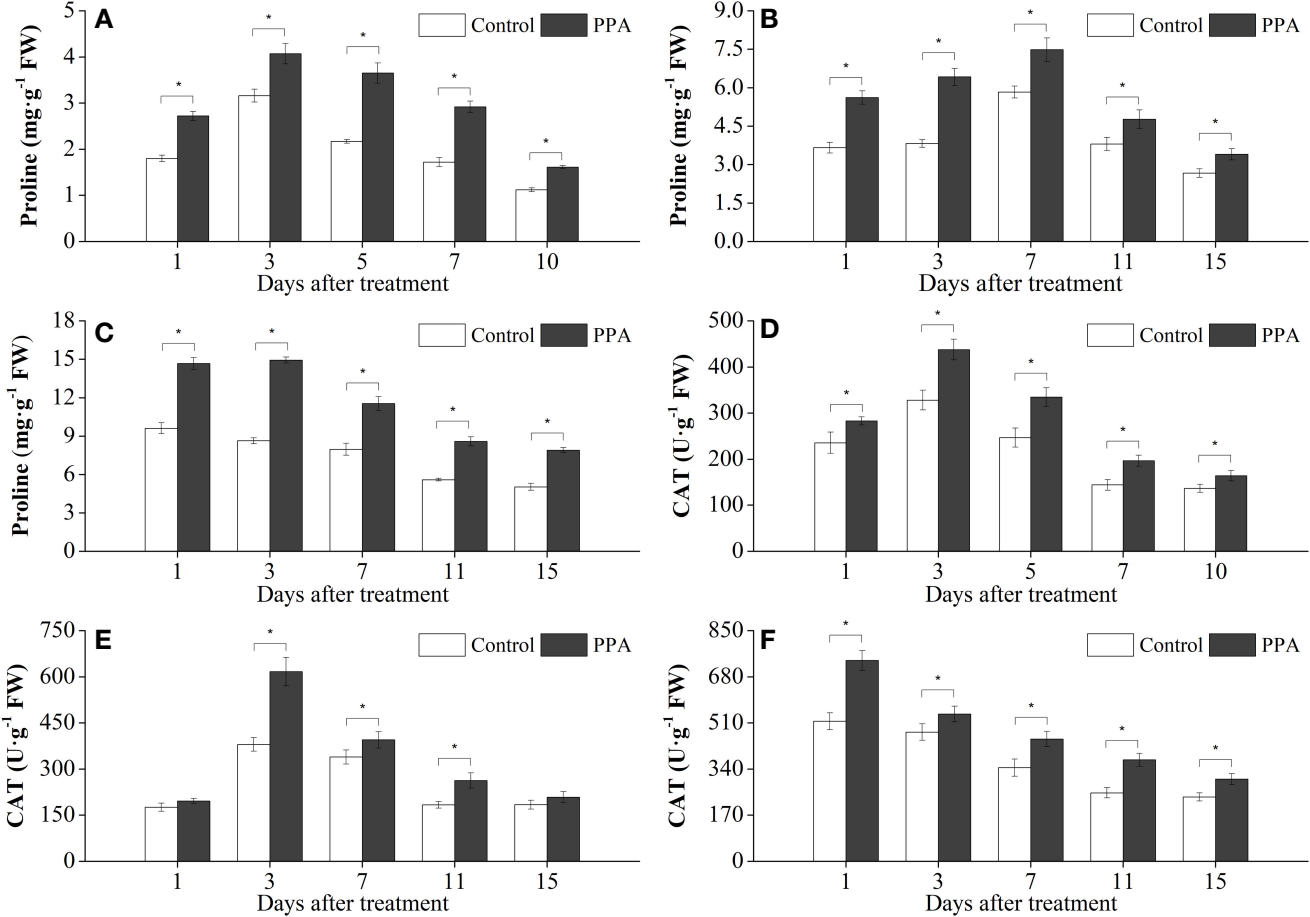
Effects of PPA on proline (**A**, SDS; **B**, MDS; **C**, LDS) and CAT (**D**, SDS; **E**, MDS; **F**, LDS) in pepper (*C. annuum*) leaves under drought stress. Asterisk denotes significant difference to corresponding control at P < 0.05.

Exogenous PPA application enhanced the activities of antioxidative enzymes (SOD, CAT, and POD) in pepper leaves under DS (**Figures 4**, **5**). POD activities were markedly (*P*< 0.05) improved 13.50% - 39.25% (from day 1 to day 7) by PPA under SDS in comparison with control (**Figure 4A**). PPA also obviously (*P*< 0.05) enhanced the POD activities during the 15 days by 21.26% - 58.30% under MDS (**Figure 4B**), and 28.64% - 65.78% under LDS (**Figure 4C**), respectively. Furthermore, PPA significantly (*P*< 0.05) elevated SOD activities under LDS (with increase rates of 16.50% on day 1), MDS (increasing by 26.40% on day 1 and 17.39% on day 11), and SDS (going up 21.43% to 36.64% from day 1 to day 10), respectively (**Figures 4D–F**). Interestingly, CAT activities in the pepper leaves treated with PPA were also higher (*P*< 0.05) than those of the control, which were increased by 13.91% - 48.13% and 20.05% - 36.08% during LDS and SDS periods, respectively (**Figures 5D, F**). During the MDS period, the CAT activities were significantly (*P*< 0.05) increased by 16.44% to 62.32% (from day 3 to day 11) (**Figure 5E**).

### 3.5 Effect of PPA on free proline in pepper seedlings under DS

Compared with the control, exogenous PPA significantly (*P*< 0.05) boosted the free proline content in the pepper leaves under LDS, MDS, and SDS by 44.61% - 72.92%, 25.53% - 68.06% and 28.80 - 69.77%, respectively (**Figures 5A–C**).

### 3.6 Effects of PPA on soluble sugar and soluble protein in the pepper seedlings under DS

**Figure 6:**
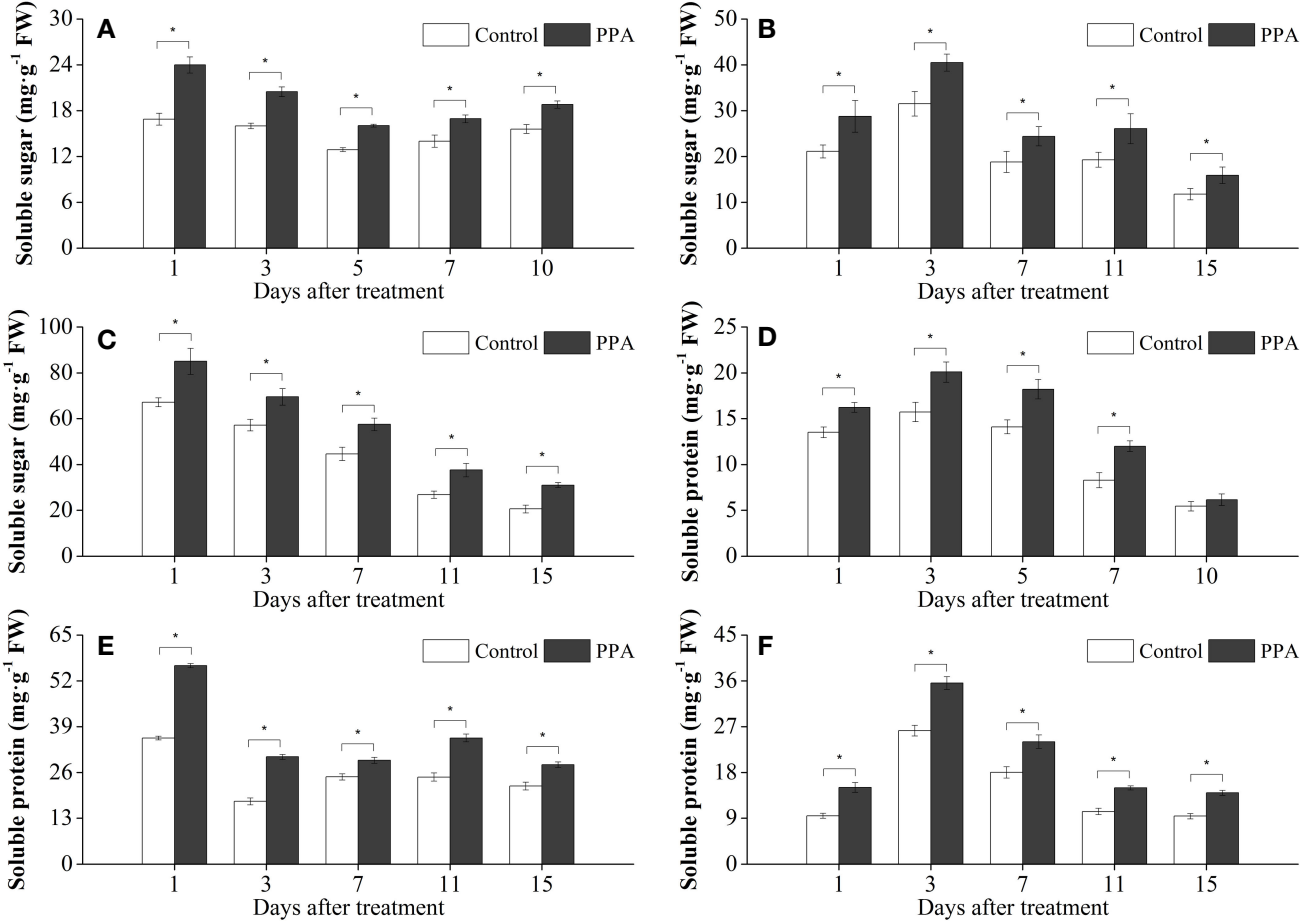
Effects of PPA on soluble sugar (**A**, SDS; **B**, MDS; **C**, LDS) and soluble protein (**D**, SDS; **E**, MDS; **F**, LDS) in pepper (*C. annuum*) leaves under drought stress. Asterisk denotes significant difference to corresponding control at P < 0.05.

As shown in **Figure 6**, PPA was associated with increases in soluble sugar and soluble protein in the pepper seedlings under drought conditions. In comparison with the control, the contents of soluble sugar were notably (*P*< 0.05) enhanced by 21.57% - 50.22% (LDS), 28.49% - 36.13% (MDS), and 20.51% - 41.98% (SDS) throughout the drought stress period (**Figures 6A–C**). Analogously, the soluble protein contents were observably (*P*< 0.05) increased by 20.13% - 44.63% over control during the SDS period except on day 10 (**Figure 6D**). Within the LDS and MDS periods, the soluble protein contents in the PPA-treated pepper leaves were significantly (*P*< 0.05) increased by 33.46% - 58.74% and 27.20% - 70.85%, respectively (**Figures 6E, F**).

## 4 Discussion

PPA plays an important role in alleviating plant growth inhibition and damage caused by DS. In general, the leaf wilting index (LWI) directly reflects the wilting degree of leaves, and its value is positively related to the tolerance of crops for DS (Wang et al., 2015). RWC represents the tissue moisture of crops, and its reduction means that the crops have suffered drought (Malika et al., 2019). In addition, MDA is the final product of membrane lipid peroxidation caused by ROS and acts as a signal of cell membrane damage (Gao et al., 2020). In this work, pepper seedlings were pretreated with PPA and then placed in DS, and it was found that LWI and RWC values were significantly raised, while the MDA content was markedly decreased, indicating that PPA could induce resistance to DS in pepper seedlings.

Obviously, the effect of PPA can be confirmed by improving the morphology of pepper. Normally, once plants are placed under DS, their most intuitive influence is morphological damage (Gupta et al., 2014). In this work, we found that exogenous PPA treatment promoted the plant height, root-shoot ratio, and seedling index of pepper plants under LDS, MDS, and SDS, especially the seedling index, which was significantly increased by PPA. These results are in accord with the effect of other PGRs on the growth of drought-stressed plants. For instance, exogenous spermidine increased the root-shoot ratio of maize (*Zea mays*) seedlings under DS (Li et al., 2018). It has been reported that 50%-80% of Pn-related leaf photoassimilates are required to meet the requirements of plant non-photosynthetic organs (Ahmadi Lahijani et al., 2018). The Pn of pepper leaves under DS was also enhanced by PPA. Therefore, the increase in PPA-mediated seedling index indicated that PPA might promote the transport of photoassimilates to the whole plant. Similarly, PPA could promote the transport of more photoassimilates to the underground part of plants, resulting in an increase in the root-shoot ratio. The underground part of plants plays a key role in absorbing water from the soil to maintain balance between transpiration and hydration (Li et al., 2018). Interestingly, in this work, the RWC of pepper leaves treated with PPA was significantly increased under drought conditions, which may be associated with the PPA-induced increase in root-shoot ratio, which also makes the plants more adaptable to drought. However, this inference needs to be further studied from a molecular perspective.

The reason why PPA improves the tolerance of plants to drought may be associated with the improvement of PPA-mediated antioxidant activity, Pn and osmotic substance accumulation. Firstly, when plants are subjected to drought, reactive oxidative species (ROS) are increased in plants (Min et al., 2019), including singlet oxygen, superoxide anion, hydrogen peroxide, and hydroxyl radicals (Etesami and Jeong, 2018), leading to membrane disruption, enzyme dysfunction, and protein oxidation and aggregation (Wang et al., 2019). The strategy for plants to resist or repair damage caused by ROS can be achieved by promoting the activity of enzymatic antioxidants (such as SOD, POD, and CAT, etc.) or non-enzymatic antioxidants (carotenoids, non-protein amino acids, and phenolic compounds, among others) (Chavoushi et al., 2019). Of course, free proline is a non-enzyme scavenger for free radicals, which can protect membranes and various macromolecules like enzymes and proteins (Xiao et al., 2008). Our research revealed that PPA promoted the content of free proline and the activities of CAT, POD, and SOD in pepper leaves under LDS, MDS, and SDS, leading to the reduction of ROS. At the same time, we observed that the MDA content of pepper leaves subjected to PPA treatment was decreased, which also verifies that ROS in drought-stressed pepper was reduced. Therefore, the enhancement of tolerance to drought in PPA-treated pepper seedlings may be linked to the activation of enzymatic and non-enzymatic antioxidant systems by PPA.

Secondly, stomata close during drought periods to limit water loss by evapotranspiration, which directly causes the reduction of Gs, further decreases the Ci, Tr, and Pn, and then affects the Ls and WUE in plants (Bhusal et al., 2019). At present, the judgment of the decline in the photosynthetic rate of plant leaves depends on the trends of Ls and Ci. In other words, the stomatal factor seems to mainly limit the photosynthetic rate with the decrease of Ci and the increase of Ls; in contrast, non-stomatal factors chiefly limit the inhibition of the photosynthetic rate as Ci increases and Ls decreases (Mantlana et al., 2008). In the present study, PPA improved Ci in pepper leaves under LDS and MDS but reduced the values of Ls. Undoubtedly, Ci in untreated pepper leaves under LDS and MDS may be reduced; on the contrary, Ls may be increased. Based on the tendency of changes in Ci and Ls, we speculated that the photosynthetic rate of the pepper leaves facing drought was primarily controlled by stomatal factors. Fortunately, Pn in PPA-treated pepper leaves under LDS and MDS was significantly enhanced, attributing to the PPA-mediated increase in Ci and Tr, especially the PPA-mediated increase in Gs. Besides, chlorophyll plays a central role in the photosynthetic light reactions, whose degradation is associated with the production of reactive oxygen in the leaves caused by droughts (Liang et al., 2018). In this study, the chlorophyll content in PPA-applied pepper leaves increased under three different degrees of drought. Meanwhile, the CAT, POD, and SOD activities in pepper leaves under LDS and MDS were improved by PPA. Thus, PPA-mediated enhancement of Pn in pepper leaves under LDS and MDS may also be related to the elimination of ROS and the protection of chlorophyll. In addition, PPA improved the WUE of pepper leaves under LDS and MDS, indicating that drought-stressed pepper seedlings modified the hydraulic structure of leaves due to the intervention of PPA, which results in the formation of efficient water use mechanisms and high Pn. In this work, the LWI of pepper leaves treated with PPA was significantly higher under DS, indicating that most of the leaves that suffered drought remained apparently normal. These results demonstrated that PPA-applied pepper plants can maintain more favorable photosynthesis, respiration, and transpiration compared to untreated pepper plants under drought.

Thirdly, the accumulation of osmotic substances such as soluble sugar, soluble protein, and proline is one of the adaptive solutions for plants under DS (Chavoushi et al., 2020). These osmolytes play an important role in holding the moisture inside tissues as well as absorbing water from the external environment, thereby ultimately maintaining the normal physiological and biochemical activity of the cells (Cheng et al., 2018). This work showed that the application of PPA was closely associated with increases in soluble sugar, soluble protein, and proline in pepper leaves under drought conditions. Especially the accumulation of these three osmotic substances was obviously increased in the pepper leaves under SDS conditions, this is similar to the results of Gao et al. (2020), which means that plants need more water to overcome the damage caused by DS. Correspondingly, this statement has been corroborated; that is, this study revealed that the RWC of pepper leaves treated with PPA was significantly improved. Thus, PPA can better maintain the water required for drought-stressed pepper seedlings by promoting the accumulation of osmotic substances.

PPA has excellent application value in field agricultural production. This study found that PPA can clearly alleviate the growth inhibition and damage to pepper seedlings caused by drought. Applying PPA (133.3 mg·L^−1^) to crops twice can produce preferable efficacy. In addition, three years of field trials have shown that PPA (133.3 mg·L^−1^) can also increase the yield of pepper fruits (Zhang et al., 2017; Wu et al., 2018; Lu et al., 2023). Therefore, PPA exerts a variety of beneficial effects in field applications.

## 5 Conclusion

Exogenous PPA can induce DS resistance in pepper seedlings, reducing the damage that DS causes to peppers. A dosage of 133.3 mg·L−1 of PPA applied twice can considerably increase the drought resistance of peppers. The PPA-mediated efficacy may be linked to the improvement of multiple physiological processes, including antioxidant activities, photosynthesis, and the accumulation of osmotic substances. PPA can help peppers adapt to drought and is worthy of widespread use in pepper production.

## Data availability statement

The original contributions presented in the study are included in the article/supplementary material. Further inquiries can be directed to the corresponding authors.

## Author contributions

XL: Conceptualization, Methodology, Data curation, Software, Writing - original draft, Writing-review & editing. QW: Conceptualization, Investigation, Data curation. KN: Investigation. HW: Data curation, Validation. GC: Writing-review & editing, Funding acquisition. JW: Writing-review & editing, Funding acquisition. ZM: Funding acquisition, Project administration, Data curation, Validation, Supervision. All authors contributed to the article and approved the submitted version.
